# *Galleria mellonella -* a novel infection model for the *Mycobacterium tuberculosis* complex

**DOI:** 10.1080/21505594.2018.1491255

**Published:** 2018-08-01

**Authors:** Yanwen Li, John Spiropoulos, William Cooley, Jasmeet Singh Khara, Camilla A Gladstone, Masanori Asai, Janine T Bossé, Brian D Robertson, Sandra M Newton, Paul R Langford

**Affiliations:** a Section of Paediatric Infectious Diseases and Allergy, Department of Medicine, Imperial College London, London, UK; b Department of Pathology, Animal and Plant Health Agency, Addlestone, UK; c Department of Pharmacy, National University of Singapore, Singapore; d MRC Centre for Molecular Bacteriology and Infection, Department of Medicine, Imperial College London, London, UK

**Keywords:** *Galleria mellonella*, mycobacteria, tuberculosis, *Mycobacterium bovis* BCG *lux*, granuloma, infection model, haemocytes, host-pathogen interactions, *Mycobacterium tuberculosis* complex

## Abstract

Animal models have long been used in tuberculosis research to understand disease pathogenesis and to evaluate novel vaccine candidates and anti-mycobacterial drugs. However, all have limitations and there is no single animal model which mimics all the aspects of mycobacterial pathogenesis seen in humans. Importantly mice, the most commonly used model, do not normally form granulomas, the hallmark of tuberculosis infection. Thus there is an urgent need for the development of new alternative *in vivo* models. The insect larvae, *Galleria mellonella* has been increasingly used as a successful, simple, widely available and cost-effective model to study microbial infections. Here we report for the first time that *G. mellonella* can be used as an infection model for members of the *Mycobacterium tuberculosis* complex. We demonstrate a dose-response for *G. mellonella* survival infected with different inocula of bioluminescent *Mycobacterium bovis* BCG *lux*, and demonstrate suppression of mycobacterial luminesence over 14 days. Histopathology staining and transmission electron microscopy of infected *G. mellonella* phagocytic haemocytes show internalization and aggregation of *M. bovis* BCG *lux* in granuloma-like structures, and increasing accumulation of lipid bodies within *M. bovis* BCG *lux* over time, characteristic of latent tuberculosis infection. Our results demonstrate *that G. mellonella* can act as a surrogate host to study the pathogenesis of mycobacterial infection and shed light on host-mycobacteria interactions, including latent tuberculosis infection.

## Background

*Mycobacterium tuberculosis* is a highly successful human pathogen that currently infects a third of the world’s population, causing 9 million new cases of tuberculosis and 1.5 million deaths annually [1]. The close association of tuberculosis with the human immunodeficiency virus (HIV) pandemic, and the rising problem of multi-drug and extreme drug resistance have created difficulties for tuberculosis control programs worldwide [2,3]. There is thus an urgent need for a greater understanding of host and *M. tuberculosis* interactions, improved vaccines to prevent infection, and novel therapies to shorten the duration of treatment and target latent tuberculosis infection (LTBI), however this has in part been hampered by the current inadequate, costly and time-consuming *in vivo* infection models for tuberculosis. Animal models currently used in tuberculosis research include non-human primates (e.g. macaques), guinea pigs, rabbits, cattle, pigs, mice and zebrafish [4]. However working with such animals is difficult as the pathogenesis and progression of *M. tuberculosis* infection is complex and there is no single animal model that mimics all the aspects of pathogenesis in humans [5,6]. Mice are the most commonly used species (because of cost, availability of inbred lines, reproducibility of infection and availability of immunological reagents) [7], but granulomas and regions of hypoxia characteristic of LTBI, are not commonly seen in the lungs and no single mouse tuberculosis model can reflect the mechanistically and morphologically diverse forms of human tuberculosis [8]. The animal models most closely resembling human *M. tuberculosis* infection are non-human primates e.g. the macaque [9,10]. However ethical, cost, and practical considerations limit widespread use [6]. To overcome these issues there is an urgent need for a rapid, well-characterized, low-cost, high-throughput model that mimics the key features of *M. tuberculosis* pathogenesis to further understand host-pathogen interactions, that can also be used to screen novel drug candidates and assess the efficacy of vaccine candidates in early stage development.

*Galleria mellonella* insect larvae have increasingly been used as a surrogate to study host-pathogen interactions in a range of micro-organisms including Gram-positive and Gram-negative bacteria and fungi [11–14], and as a rapid model to screen novel antimicrobial drug candidates [15]. *G. mellonella* has a wide range of advantages that have made it a successful infection model. These include its sophisticated innate immune system, comprised of cellular and humoral defenses, that shares a high degree of structural and functional similarity to that of vertebrates [16,17], and its ability to discriminate between bacterial and fungal pathogens as identified by the repertoire of immune defense peptides [18]. *G. mellonella* are also easy to manipulate due to their large size (2–3 cm), allowing easy infection and collection of tissue/haemolymph for analysis. They are easy to maintain at 37°C making them well-suited to studying human pathogens, and precise infection studies can be performed by injection without anaesthesia for testing the efficacy of small quantities of antimicrobial agents. In addition, *G. mellonella* are ethically more acceptable than the use of vertebrates meaning larger group sizes can be used to enhance reproducibility [11,15,19,20].

Here we report the use of *G. mellonella* as a novel infection model for members of the *M. tuberculosis*-complex. Due to safety and practical considerations when using *M. tuberculosis* as an experimental organism, we have carried out this study using the non-pathogenic, luminescent reporter vaccine strain, *M. bovis* BCG *lux* [21], which has been well characterized and validated in a wide range of *in vitro* and *in vivo* models and clinical studies (e.g. liquid broth, human whole blood and cells, mouse) and has the advantage of faster measurement of mycobacterial viability [22–32]. Over a time-course we have assessed the survival of *G. mellonella* larvae in response to infection with different inocula of *M. bovis* BCG *lux*, and monitored the survival of *M. bovis* BCG *lux* by measuring bioluminescence and correlating with CFU. Furthermore we have undertaken histopathology staining and transmission electron microscopy (TEM) analysis of *M. bovis* BCG *lux* infected *G. mellonella* to look at bacteria inside phagocytic haemocytes and study the pathogenesis and progression of mycobacterial infection. Our results demonstrate that this mycobacteria-*G. mellonella* infection model can be used to study mycobacterial pathogenesis, particularly in the context of granuloma formation, and has the potential to be used in the assessment of novel vaccine candidates and anti-mycobacterial agents. The availability of this novel mycobacterial infection model also has the potential to significantly reduce the number of experimental animals used in some areas of tuberculosis research.

## Results

### Survival analysis of G. mellonella following infection with M. bovis BCG lux

*G. mellonella* survival (n = ≥ 10 per group), in response to differing inocula of *M. bovis* BCG *lux* infection (up to 2 × 10^7^ CFU per larva) was determined over a 96 h period. Larvae that did not show any movement in response to touch were considered dead.

**Figure 1.**
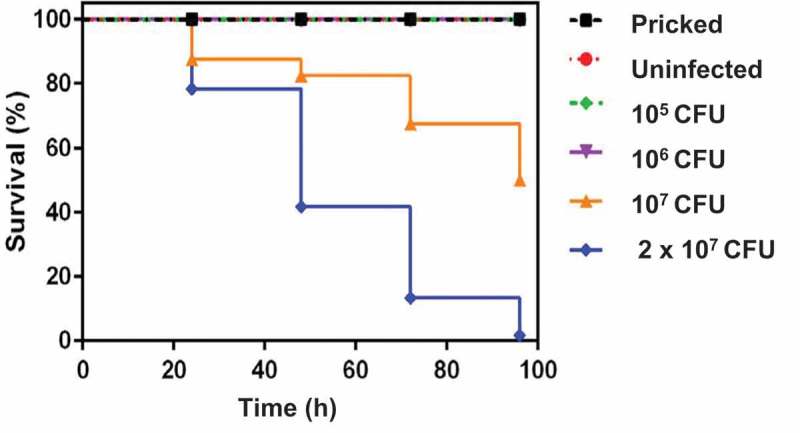
Effect of varying inoculum dose of *M. bovis* BCG *lux* on the survival of *G. mellonella* larvae following incubation at 37°C for 96 h. Healthy larva (n = ≥ 10 per group) infected with differing inocula of *M, bovis* BCG *lux* (up to 2 × 10^7^ CFU per larva), were examined for survival by response to touch at 24 h intervals over a period of 96 h. Control groups (n = 10 larvae per group) included an uninfected group inoculated with 10 µl PBS-Tween 80, and a ‘pricked’ larval group (empty needle injection). Larvae were incubated at 37°C in the dark and monitored on a daily basis. Larvae that did not show any movement in response to touch were considered dead. Data are pooled from at least two independent experiments with a minimum of 10 larvae per group per run.

*G. mellonella*, infected with doses of 1 × 10^5^ or 1 × 10^6^ CFU *M. bovis* BCG *lux*, survived 100% (Figure 1 and Supplementary Table 1) as did the ‘pricked’ (empty needle injection) and ‘uninfected’ (PBS-Tween 80) control groups. This demonstrated that neither the needle injection nor the 0.05% Tween 80 used to prevent mycobacterial clumping, had any effect on *G. mellonella* survival. At higher doses of *M. bovis* BCG *lux* (1 x 10^7^ and 2 × 10^7^ CFU), a concentration-dependent effect on survival was observed. 50% *G. mellonella* survival was observed using a dose of 1 × 10^7^ CFU *M. bovis* BCG *lux* per larva at 96 h, however there was less than 1% survival using a dose of 2 × 10^7^ CFU per larva at this time-point. A time period of 96 h was used to measure larval survival, as at approximately 1 week of incubation at 37 C some of the larva in the ‘pricked’ and ‘uninfected’ groups started to pupate as they entered the next stage of the life cycle. Other observations of mycobacterial infected larvae compared to uninfected larvae over time, were a slight darkening in color and reduced flexibility and size, characteristic of the organism wasting.

### M. bovis BCG lux survival in G. mellonella over time

The survival of *M. bovis* BCG *lux* (dose, 1 × 10^7^ CFU) within *G. mellonella* was determined over a 2 week time-course to further validate *G. mellonella* as an infection model for mycobacteria. Here we investigated the recovery of *M. bovis* BCG *lux* by luminescence measurement over time (0, 24, 48, 72, 96, 168 and 366 h) from homogenized larvae (n = 5 per time-point) to assess the ability of the immune system of *G. mellonella* to control mycobacterial growth. Only live, non-melanized larvae were used for homogenization.

**Figure 2.**
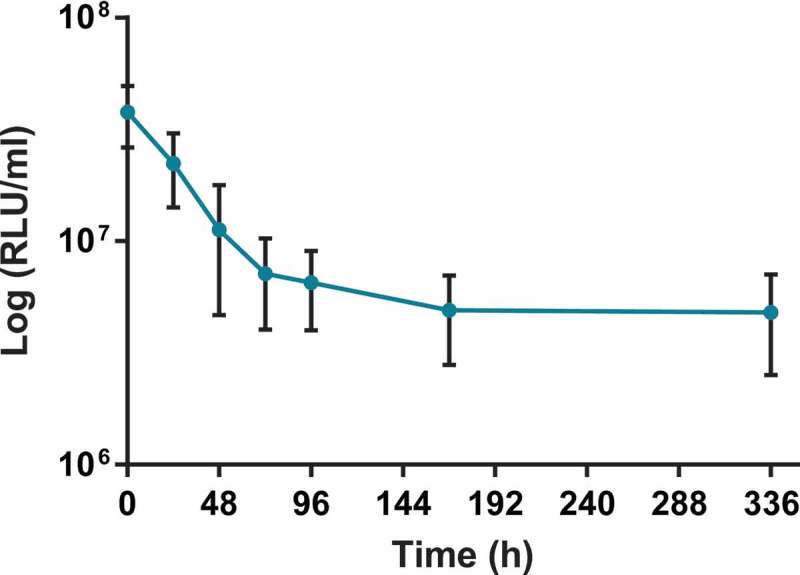
Recovery of *M. bovis* BCG *lux* (dose 1 × 10^7^ CFU per larva) by luminescence measurement (RLU/ml) over a 2 week time-course from homogenized infected larvae. At least 30 larvae were infected with 10 µl of 1 × 10^7^ CFU *M. bovis* BCG *lux*. The survival of *M. bovis* BCG *lux* within live *G. mellonella* was determined by luminescence measurement over time (24, 48, 72, 96, 168, 366 h) from the lysates of 5 homogenized larvae to assess the ability of the immune system of *G. mellonella* to control mycobacterial growth. Data are pooled from three independent experiments with 5 larvae per time-point per run. Error bars show standard deviation of the mean.

When *G. mellonella* were inoculated with a dose of 1 × 10^7^ CFU per larva there was approximately a 1 log reduction in *M. bovis* BCG *lux* luminescence between 0 and 168 h, with a further steady decrease in bacterial numbers until the end of the time-course at 366 h (Figure 2). When we measured live *G. mellonella* uninfected crude extracts for background luminescence, very low levels were detected ranging from 5000–8000 RLU/ml indicating this did not have any impact on mycobacterial luminescence.

In addition to the luminescence measurement of mycobacteria, we also plated homogenized samples (n = 4 larva per time-point) for CFU quantification at the corresponding time-points up to 168 h on to Middlebrook 7H11 agar, containing hygromycin as an antibiotic selection marker and piperacillin which inhibited the growth of contaminating *G. mellonella* bacterial flora. Contamination from the normal bacterial flora of *G. mellonella* has been observed in other studies [33,34] but a recent paper by Entwistle & Coote [35] demonstrated that the antibiotic piperacillin was able to inhibit *G. mellonella* contaminating bacterial flora but did not inhibit the growth of non-tuberculous mycobacteria. We undertook a minimum inhibitory concentration (MIC) assay to assess the inhibitory concentration of piperacillin against *M. bovis* BCG *lux* at doubling concentrations ranging from 1280 – 10 µg/ml. The MIC of piperacillin against *M. bovis* BCG *lux* was 160 µg/ml. A concentration of 10 µg/ml was found to inhibit the growth of contaminating *G. mellonella* bacterial flora and hence was added to agar plates for *M. bovis* BCG *lux* CFU quantification. We found that the RLU:CFU ratio was consistent over the time-course with an average ratio of 4:1 (range 2:1 to 5:1) (data not shown).

### Histopathological analysis of M. bovis BCG lux infection of G. mellonella larvae

Granulomas in the human host, are highly dynamic and characteristic cellular structures that are the hallmark of tuberculosis and not only contain and control infection but also provide a niche for mycobacteria to survive. However, not all animal models form such structures in response to mycobacterial infection.

*G. mellonella* has an innate immune system comprised of cellular and humoral defenses including phagocytosis of pathogens by haemocytes (a similar cell type to human macrophages and neutrophils) [36] and the production of antimicrobial peptides, complement-like proteins and reactive oxygen and nitrogen species for killing ingested pathogens, all found within the haemolymph of *G. mellonella* [16]. Here we undertook both histopathology and TEM to follow the progression of *M. bovis* BCG *lux* infection, assess the impact of infection on larval tissues and determine whether granulomas are induced in this model of infection.

**Figure 3.**
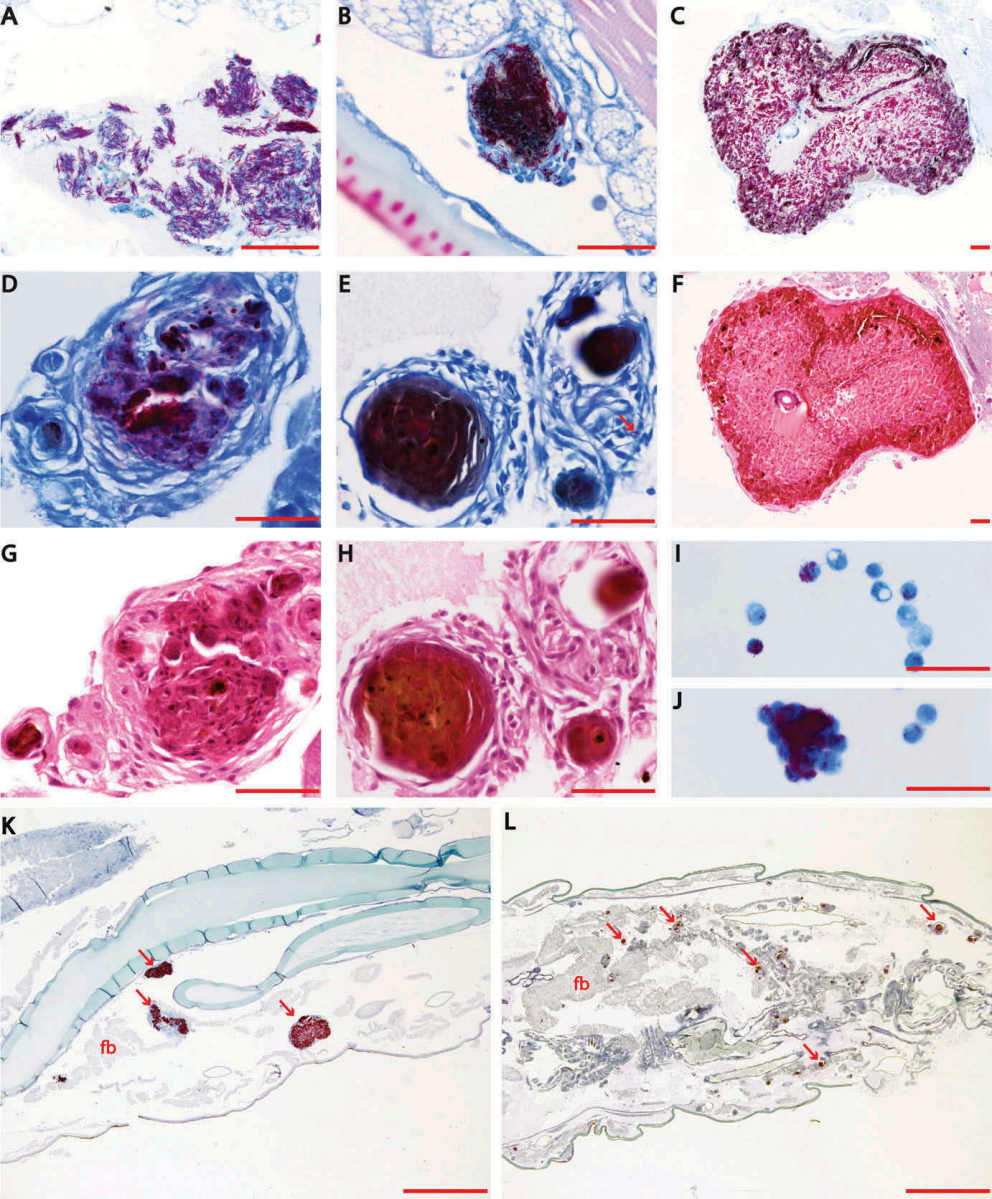
Histopathological analysis of *M. bovis* BCG *lux* infection of *G. mellonella* larvae (1 x 10^7^ CFU per larva) over a time-course of 2 weeks. Both *M. bovis* BCG *lux* infected and uninfected (pricked and PBS challenged) larvae (n = 3 per group) were fixed by injecting 100 µl of buffered formalin with an insulin syringe in to the last left pro-leg at each time-point (0, 24, 48, 72, 96 h for uninfected and infected larvae, and additional time-points of 144 and 336 h were also included for infected larvae). Samples were embedded in paraffin wax, sectioned at 4 µm and then stained with haematoxylin and eosin (H&E) and Ziehl-Neelsen (ZN) stain for acid fast bacteria. Individual bacilli are discernible arranged loosely (A, 24 h post challenge) or organized in early granuloma-like structures (B, 24 h post challenge). Large well-defined granuloma-like structure containing individual bacilli (C, 72 h post challenge) and a granuloma-like structure (D, 96 h post challenge) with individual bacilli and amorphous acid fast-material (arrow). At subsequent stages of infection the acid fast material within the granuloma like structures was predominantly amorphous (E, 366 h post challenge) although individual bacilli may still be detected (arrow). Within the granuloma-like structures host cells are undergoing necrosis (F-H) and brown pigment may be associated with the necrotic areas (H). Haemolymph smears show that the bacilli are co-localized with individual cells (I) or cell aggregates (J) which most likely represent granuloma-like structures. Sections K and L (72 and 144 h post infection respectively) provide lower magnification images showing that the granulomas are dispersed in the haemocoel and they can be associated with the fat body (arrows indicate some characteristic granulomas). In Section K, the granulomas are similar to the ones shown in Sections B and C where individual bacilli can be identified. In Section L the granulomas are similar to the compact granulomas shown in Section E. Sections A-E, K, L, and smears I and J are stained with ZN. Sections F-H are stained with H&E and demonstrate the same structures shown in C-E respectively in adjacent planes (serial sections). Scale bar in images A-J represents 50 µm; in images K and L it represents 1000 µm; fb, fat body.

Histopathology analysis of sectioned larvae infected with *M. bovis* BCG *lux*, and stained using both H&E and ZN (acid-fast), demonstrated that at 24 h post-challenge, rod-shaped acid-fast bacilli (stained pink) were visible and many were co-localized with host cells (stained blue) (Figure 3(A)), presumably haemocytes. We also observed isolation and compartmentalization of bacilli within granuloma-like structures (Figure 3(B-D)). At 48 h post-challenge acid-fast amorphous material began to appear, most likely representing the by-product of destroyed acid-fast bacteria (Figure 3(D)). At subsequent time-points the acid-fast amorphous material within the granuloma-like structures increased, whilst individual bacilli were observed less frequently. By 168 h, the amorphous acid-fast material was the main structure associated with the granuloma-like structures, although occasionally a small number of individual bacilli were also observed (Figure 3(E)). Staining serial sections with H&E demonstrated that the bacilli in the granuloma-like structures were co-localized with host cells (Figure 3(F,G)). In the center of the granuloma-like structures necrotic host cells were evident (Figure 3(F-H)) and in some cases brown pigmented material which co-localized with the amorphous acid-fast material was detected (Figure 3(H)). Although using histology it was not possible to demonstrate that the bacilli were internalized, haemolymph smears stained with ZN appeared to demonstrate intracellular location of *M. bovis* BCG *lux* bacilli in individual host cells (Figure 3(I)) and in cell aggregates (Figure 3(J)), which have the appearance of granuloma-like structures. These structures were located in the haemocoel presumably surrounded by haemolymph, which was not possible to demonstrate in histological sections. Sometimes the granulomas were in contact with the fat body and infrequently with other internal organs (Figure 3(K,L)). No ZN positive bacilli were identified in uninfected (pricked) larvae or in larvae challenged with PBS (Supplementary Figure 1).

### Phagocytosis of M. bovis BCG lux infected haemocytes in vivo

**Figure 4.**
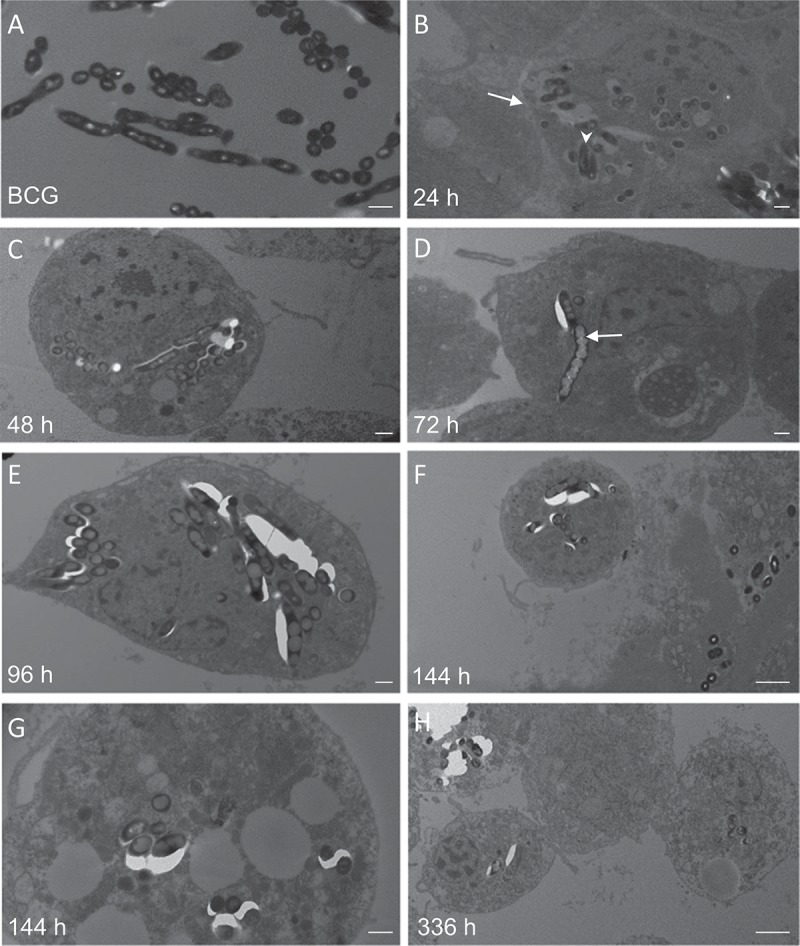
Transmission electron microscopy (TEM) of *M. bovis* BCG *lux* infected haemocytes (dose 1 × 10^7^ CFU per larva) *in vivo from G. mellonella* over a time-course from 24 h to 336 h. Haemolymph was extracted from the haemocoel of *G. mellonella* (n = 10) at 24 h intervals post-infection by puncturing the larvae in the last left pro-leg using a sterile needle. Haemolymph was pooled, processed and analyzed using a Tecnai bioTWIN transmission electron microscope. *M. bovis* BCG *lux* inoculum alone served as a control (A). Further figures (B-H) show the haemolymph contains haemocytes (arrow, B) and the time-course study showed the ultrastructure of initial internalization of BCG *lux* into cells at 24 h (arrowhead, B). From 24 h to 366 h, aggregation of bacilli, typical to that found in granulomas was evident. TEM confirmed that over the time-course the bacteria invade or are phagocytosed by haemocytes and multiply within to form aggregations of bacilli, typical of those found in granulomas. There is also an increase in lipid material within *M. bovis* BCG *lux* during the time-course (arrow, D). Scale bar represents 500 nm (A-E, G) and 2 µm (F, H).

To assess phagocytosis of *M. bovis* BCG *lux* (dose 1 × 10 ^7^ CFU) by *G. mellonella* haemocytes over a time-course of 2 weeks, TEM was undertaken on haemolymph samples extracted from infected *G. mellonella* (n = 3) at 24, 48, 72, 96, 144 and 366 h post-infection. Both *M. bovis* BCG *lux* inoculum (A) and uninfected *G. mellonella* at 96 h (n = 3) (Supplementary Figure 2(A)) served as controls. TEM (Figure 4), over the time-course, identified ultrastructurally internalized *M. bovis* BCG *lux* into cells from 24 h (Figure 4(B-H)). From 24 h to 366 h, aggregations of bacilli, typical of that found in granulomas was evident. TEM was able to confirm the histopathological findings suggesting that bacteria invade or are phagocytosed by haemocytes and multiply within to form aggregate-like structures. An increase in lipid material within *M. bovis* BCG *lux* during the time-course was also observed. Images of uninfected haemocytes, compared to *M. bovis* BCG *lux* infected haemocytes, at 96 h post-infection are shown in Supplementary Figure 2(A-B).

## Discussion

The use of invertebrates, such as *Caenorhabditis elegans*, *Drosophila melanogaster* and *G. mellonella*, as models for infection studies have been widely reported as they are considerably cheaper, ethically less contentious than mammals, and although insects lack an acquired immune system they do possess a complex innate immune system [37]. Furthermore *D. melanogaster* has been used as an infection model for *M. abscessus* and *M. marinum*, particularly to study the pathogenesis and metabolic consequences of mycobacterial infection [38–41]. The larvae of *G. mellonella* have been widely used to study host-pathogen interactions for a range of microbes, including fungi, bacteria and viruses [11–13,42–45], but little has been reported using mycobacteria [35,46–48], for which new *in vivo* infection models are greatly needed. Here we have demonstrated that this simple invertebrate model shows promise as a novel infection model for the *M. tuberculosis* complex.

Over 1000 articles have been published on the use of *G. mellonella* as a surrogate host infection model for studying the pathogenesis of micro-organisms, with more than 200 publications in 2014–2015 alone [15]. Advantages of using this insect larva include, no ethical implications, wide availability of larvae from commercial suppliers, low cost, simple housing and easy maintenance at 37°C, which also mimics the human host, large size (20–30 mm) allowing easy manipulation without the need for anaesthesia, precision injection into the haemocel through the last pro-leg, and the availability of host tissue/haemolymph for experimental analysis [11,15,19,20]. In addition, a good correlation has been established between the pathogenicity of several micro-organisms in *G. mellonella* with vertebrate models of infection [49]. Moreover, *G. mellonella* has an innate immune system, comprised of cellular and humoral defenses, that share a high degree of structural and functional similarity to that of vertebrates [16], and has been shown to discriminate between bacterial and fungal pathogens as judged by the repertoire of immune defense peptides [18]. A transcriptomic study of *G. mellonella* larvae found a large repertoire of immune effectors, many shared with mammals [50]. Hence bacteria can elicit an immune response in *G. mellonella* that is species-specific and is attractive for studying the pathogenesis of micro-organisms. This model also has the benefits of allowing infection experiments to be undertaken in a controlled manner with precise time-course measurements, analysis of changes in host-pathogen transcriptome, proteome and biochemistry, and far greater group sizes and numbers of replicates (n = 20 minimum per group) compared to animal studies (e.g. mouse n = 5 per group), enhancing reproducibility.

In this study we set out to evaluate the use of *G. mellonella* as an infection model for *M. bovis* BCG *lux*, a luminescent derivative of the vaccine strain that has the advantages of faster measurement of mycobacterial viability (luminescence requires co-factors only found in living metabolically active cells) compared to the slow growing *M. tuberculosis* with a doubling time of approximately 24 h [21,22,24]. Additionally, *M. bovis* BCG *lux* requires containment level (CL) 2, rather than CL3 laboratories. This organism has been very well-characterized and utilized in a wide range of clinical and non-clinical studies [21,24,27–32,51]. For example, differences in *M. bovis* BCG *lux* RLU measurements have been shown to reflect changes in the immune response in human whole blood and cellular models, including differences between tuberculin-positive and -negative individuals [23], changes induced by HIV infection and following antiretroviral treatment [25,26], changes in mycobacterial growth limitation induced by vaccines [24] and Vitamin D supplementation *in vitro* and in a clinical trial [29–31], and differences in gene expression in immune biological pathways compared to *M. tuberculosis* [32]. In addition we have also evaluated luminescent mycobacteria in the mouse model [27]. Hence the use of *M. bovis* BCG *lux* in *in vivo* studies as a model organism for *M. tuberculosis* lends further benefit to *in vivo* assays using *G. mellonella*.

To characterize the infection model we first assessed the virulence of *M. bovis* BCG *lux* in *G. mellonella* using a range of doses, and determined survival of both host and mycobacteria over a 96 h time-course of infection. All *G. mellonella* survived at infection doses of 1 × 10^5^ and 1 × 10^6^ CFU of *M. bovis* BCG *lux*, whereas at higher doses a concentration-dependent effect was observed. A dose of 1 × 10^7^ CFU was therefore selected for further time-kill, histopathology and TEM studies and would be suitable for testing drug treatments using this model.

The observation of ‘wasting’ in mycobacteria infected larvae, compared to uninfected, is consistent with the clinical feature of tuberculosis in humans and animals and warrants further investigation. This has also been described by Dionne and Schnieder in the *D. melanogaster* model of *M. marinum* infection [39].

Due to differences between bacterial species, strains, growth rates, pathogenicity and inocula used to infect *G. mellonella* and also time-points for larval survival measurements, comparing larval survival between bacterial species is difficult with, ideally, side by side experiments under the same conditions being carried out. However, at the 96 h time point with an equivalent dose of 1 × 10^7^ CFU of *M. bovis* BCG *lux* or *M. fortuitum* NCTC 10394 [35], 50% and no larvae respectively, survived. Comparative survival may reflect differences in the mechanism(s) of pathogenicity or that *M. fortuitum* NCTC 10394 is a faster growing species.

We also assessed survival of *M. bovis* BCG *lux* over time-course of 366 h using a dose of 1 × 10^7^ CFU per larva and observed approximately a 1 log reduction in luminescence between time-points 0 and 168 h post-infection which continued to steadily decrease until the end of the time-course, indicating persistent viable mycobacterial infection. Whilst *G. mellonella* larvae do not possess an adaptive immune system they do display an innate immune system similar to humans with many analogous functions including, phagocytosis of pathogens by haemocytes [17,34,52–54], a similar cell type to human macrophages and neutrophils [36]. They also produce antimicrobial peptides, complement-like proteins and reactive oxygen and nitrogen species for killing ingested pathogens, all found within the haemolymph, a biological fluid similar to mammalian blood [15,16,18]. We speculate that death of infected larvae with higher *M. bovis* BCG *lux* doses (10^7^ and 10^6^ CFU per larvae) occurs despite no increase in mycobacterial growth, as a result of an overwhelming *G. mellonella* immune response at the site of infection. Further investigation of the molecular and functional immune mechanisms and specific cells involved in suppression of mycobacterial luminescence will be the subject of future work.

Luminescence measurement has been shown to be a measure of mycobacterial metabolic activity, related to viability [21,22,24,33]. We also isolated and enumerated mycobacterial CFU from infected *G. mellonella* at the same respective time-points as luminescence measurements up to 168 h to determine the correlation between RLU and CFU in the *G. mellonella* model. *G. mellonella* homogenized larvae were plated on to Middlebrook 7H11 agar, specific for mycobacterial growth, incorporating hygromycin as an antibiotic selection marker for the *lux* reporter and piperacillin to inhibit the growth of contaminating bacterial flora from *G. mellonella.* We found the MIC of piperacillin against *M. bovis* BCG *lux* to be 160 µg/ml and whilst inhibiting the growth of *G. melonella* bacterial flora, it did not inhibit the growth of *M. bovis* BCG *lux* at the concentration used (10 µg/ml). We found that the RLU: CFU ratio for *M. bovis* BCG *lux* correlated well at each time-point over the time-course with an average RLU: CFU ratio of 4:1 which is also consistent with the RLU: CFU 3:1 ratio in liquid broth culture. These findings highlight that although luminescence is a measure of metabolic activity in *M. bovis* BCG *lux*, it correlates with CFU *in vivo* in the *G. mellonella* model over time and further highlights the benefit of using this luminescent reporter organism. Similarly the experiments of Meir *et al*. [48] showed that there was correlation between *lux* RLU and CFU with the fast growing non-tuberculous *M. abscessus* in the background of *G. mellonella*, as has also been shown with other bacteria e.g. *Enterococcus faecalis* [55].

The hallmark of tuberculosis infection in humans is the granuloma; a highly organized and dynamic cellular structure where mycobacteria reside in a non-replicating dormant state known as LTBI [6]. Several types can be found in humans including the caseous, non-necrotizing, and fibrotic granulomas [56]. However studying mechanisms underlying latency and reactivation of tuberculosis is hampered by the limitations of current animal models [57]. Furthermore the design of new drugs which penetrate granulomas to target LTBI in particular, is an urgent priority for any treatment control program [58]. Recently the zebrafish tuberculosis model, using *M. marinum*, has been used to mimic aspects of LTBI due to its small size, rapid reproduction, formation of granulomas and advanced genetic tools, making it a suitable animal model for large-scale screening of novel therapeutic agents in early-stage preclinical studies. However, zebrafish are anatomically and physiologically different to humans and lack the clinical manifestations and symptoms of tuberculosis disease and moreover they cannot be infected with the pathogenic human strain *M. tuberculosis* [4,59–62].

Granuloma-like structure formation in *G. mellonella* has been shown for the pathogenic fungal infections *Paracoccidioides lutzii* and *Histoplasma capsulatum* [34]. Hence, we undertook both histopathology staining and TEM to visually assess the progression of infection and examine the changes in host tissue and induction of granuloma-like formations in response to infection. Our data show that *M. bovis* BCG *lux* challenged larvae induce granuloma-like structures to sequester the bacteria as early as 24 h following infection, and inside these formations the bacilli lose their characteristic morphology forming amorphous acid-fast bodies by 48 h. Analysis of infected phagocytic haemocytes, extracted from *G. mellonella* larvae, show internalization and aggregation of *M. bovis* BCG *lux* in granuloma-like structures, with increasing accumulation of lipid bodies within *M. bovis* BCG *lux* over time. The accumulation of lipid bodies, in the form of triacylglycerides (TAGs), is evident in *M. tuberculosis* residing within lipid-rich foamy macrophages in human granulomas [63,64], and is proposed to serve as a source of carbon and energy representing a hallmark of persistent and non-dividing mycobacteria, labelled the drug tolerant, ‘fat and lazy’ bacilli [63,65]. It is therefore no surprise using this infection model, that mycobacteria are highly likely sourcing lipid rich material within their granuloma-like structures from their fatty *G. mellonella* host. These granuloma-like structures are formed in response to challenge with *M. bovis* BCG *lux*, which is an attenuated *M. bovis* strain. It would be of significant interest to study the response of *G. melonella* larvae to pathogenic mycobacterium (e.g. *M. tuberculosis*, *M. bovis*) as the vast majority of data in mammals derives from exposure to these species.

Whilst further investigation is required into the nature and composition of both the granuloma-like structures formed in response to *M. bovis* BCG *lux* infection, and the intracellular mycobacterial lipid bodies, our results are highly encouraging that *G. mellonella* represent an organism capable of forming such pathological features of tuberculosis, which are highly desirable for a novel tuberculosis infection model. In addition a range of mycobacterial strains, mutants classes (e.g. lipids, metabolism, persistence, stress) and reporters (e.g. luminesence and fluorescence), to assess host-pathogen interactions and characterize mechanisms of pathogenicity, could be used in this model.

In summary, this is, to our knowledge, the first report of *G. mellonella* larvae being used as a suitable infection model for a member of the *M*. *tuberculosis*-complex. Importantly we show that *G. mellonella* replicates features of the pathogenesis of tuberculosis through the induction of granuloma–like structures characteristic of LTBI, and the inclusion of lipid bodies within mycobacteria over time, characteristic of persistent infection. Further evaluation of this model has the potential to markedly reduce the use of more expensive and time-consuming animal models for evaluating mycobacterial host pathogenicity, the toxicity and efficacy of novel anti-mycobacterial agents and novel vaccine candidates *in vivo* [66]. The characterization and optimization of this model with both non-pathogenic, and pathogenic drug-sensitive and drug-resistant *M. tuberculosis* isolates will form the basis of future studies.

## Materials and methods

### M. bovis BCG lux growth conditions

*M. bovis* BCG *lux* (Montreal vaccine strain) was a kind gift from Professor Young’s lab (Imperial College London), transformed with the luminescent reporter plasmid construct pSMT1 carrying the *luxAB* genes from *Vibrio harveyi* [21]. *M. bovis* BCG *lux* were grown shaking at 220 rpm and 37°C until mid-log phase in Middlebrook 7H9 broth (Difco, MI) medium containing 0.2% glycerol (Sigma-Aldrich), 0.05% Tween 80 (Sigma-Aldrich), 10% ADC enrichment (Difco, MI) and 50 µg/ml hygromycin (Roche) as an antibiotic selection marker. Frozen aliquots were prepared in 15% glycerol and stored at −80°C until further use. Mycobacterial colony forming unit enumeration (CFU)/ml was determined by serial dilution on 7H11 agar (Difco, Detroit, MI) containing 0.5% glycerol, 10% oleic acid-albumin-dextrose-catalase (Difco, MI) enrichment and 50 µg/ml hygromycin. *M. bovis* BCG *lux* luminescence was measured after addition of decanal (Sigma) substrate (1% v/v in ethanol), as relative light units (RLU)/ml in a luminometer (Berthold Technologies) and correlated with CFU/ml counts. A ratio of 3 RLU correlated to 1 CFU in liquid broth culture as previously described [22].

### Optimization and preparation of M. bovis BCG lux inoculum

Prior to infection experiments, a vial of mycobacteria was defrosted and grown in liquid medium (as described above) to mid-log phase for 72 h with *M. bovis* BCG *lux* growth monitored by luminescent measurements on a daily basis [22]. Cultures were centrifuged at 3880 g for 10 minutes, supernatant decanted and the cell pellet was resuspended in phosphate buffered saline (PBS) (Sigma-Aldrich) containing 0.05% (v\v) Tween 80 (PBS-Tween 80) and washed a further twice. PBS-Tween 80 was found to prevent mycobacterial clumping particularly when achieving dense concentrations of mycobacterial inoculum (e.g. 10^9^ RLU/ml). The washed cell pellet was diluted in PBS-Tween 80 and the required mycobacterial inoculum measured by luminescence, resulting in a homogenous suspension. The inoculum dose was verified by plating on to Middlebrook 7H11 agar.

### In vivo virulence assays of G. mellonella infected with M. bovis BCG lux

Final instar larvae of *G. mellonella* were obtained from Livefoods Direct Ltd, Sheffield, UK and stored in wood shavings in the dark at 19°C. Healthy larvae were identified by their cream colour, without dark discolouration, the ability to right themselves if turned on their backs, a body weight of approximately 250 mg and body length of 2–3 cm, and were used in infection experiments within 3 days of delivery. For experimental use healthy larvae were counted into 25 cm Petri dishes containing a layer of filter paper. Once selected, no food was made available. Larvae displaying signs of death (no movement when touched) were discarded along with those undergoing pupation.

Prior to each experiment, larvae were surface disinfected with ethanol, and at least 10 larvae per group were injected with 10 μl of a *M. bovis* BCG *lux* suspension in PBS-Tween 80. Doses, between 1 × 10^5^ and 2 × 10^7^ CFU, were injected via the last left pro-leg into the haemocoel using a SGE 25 µl microvolume syringe. Control groups (n = 10 larvae) included an uninfected group inoculated with 10 µl PBS-Tween 80, and a ‘pricked’ larval group (empty needle injection). Larvae were maintained in petri dishes containing a base layer of filter paper at 37°C in the dark. All larvae were monitored on a daily basis and melanization and survival were recorded. Larvae that did not show any movement in response to touch were considered dead. Data are pooled from at least two independent experiments with a minimum of 10 larvae per run. Kaplan-Meier survival curves of *G. mellonella* in response to pricked, uninfected and *M. bovis* BCG *lux* infection were plotted to display percentage survival over time.

### Recovery of M. bovis BCG lux from G. mellonella over time

At least 30 larvae were infected with a 10 µl dose of 1 × 10^7^ CFU as described above. At 24, 48, 72, 96, 168 and 366 h, five live larvae per time-point (no melanization) were disinfected by wiping down with 70% ethanol and homogenized in PBS-Tween 80 using a FastPrep-24 5G (MP Biomedicals) with sterile metal beads. Mycobacterial luminescence (RLU/ml) was measured in the homogenized lysate as described above. The mean RLU/ml of *M. bovis* BCG *lux* survival in *G. mellonella* was determined over 366 h. In addition, CFU enumeration was also determined in homogenized lysate by plating serial dilutions in duplicate on to Middlebrook 7H11 agar plates containing 50 µg/ml hygromycin as an antibiotic selection marker and 10 µg/ml piperacillin (Sigma) to inhibit the growth of *G. mellonella* bacterial flora. The mean CFU/ml of *M. bovis* BCG *lux* survival in *G. mellonella* was determined at each time-point over 168 h.

To determine CFU enumeration of the inoculating *M. bovis* BCG *lux,* 100 µl of mycobacterial inoculum was plated on to Middlebrook 7H11 agar plates, containing 50 µg hygromycin.

### Minimum inhibitory concentration (MIC) assay of piperacillin

The MIC of piperacillin was determined against *M. bovis* BCG *lux*. Two-fold serial dilutions of piperacillin ranging from 1280 to 10 μg/ml were prepared in Middlebrook 7H9 broth and 100 μl of each concentration was combined with an equal volume of log-phase mycobacterial culture diluted to ~ 10^6^ CFU/ml in a 96 well plate. Growth media containing mycobacteria only was used as the positive control and growth media alone as the negative control. For each concentration and control was performed in triplicate. Plates were incubated in a rocking incubator at 20 rpm at 37 °C for 7 days. The MIC was defined as the lowest concentration at which no mycobacterial growth was observed visually or spectrophotometrically using a plate reader (Molecular Devices, UK) at an optical density 595 nm. Each MIC experiment was performed in triplicate.

To determine the concentration of piperacillin required to inhibit the growth of *G. mellonella* bacterial flora, 50 µl homogenized larvae (n = 5 larva) was plated on to Middlebrook 7H11 agar plates containing a range of concentrations of piperacillin (10, 20, 40 µg/ml). Agar plates without piperacillin were used as the positive control. Plates were incubated at 37°C for up to 2 weeks to check for growth of *G. mellonella* bacterial flora.

### Time course, histopathology and transmission electron microscopy (TEM) analysis of M. bovis BCG lux recovered from G. mellonella

The time-course study was undertaken to investigate the progress of *M. bovis* BCG *lux* infection (dose 1 × 10^7^ CFU) within *G. mellonella* infected larvae, and assess the impact of infection on *G. mellonella* internal tissue. Three larvae per time-point per group were used for histopathology analysis and at least ten larvae per time-point for TEM. The time-course ran for 96 h post-infection, with time-points at 0 h and then every 24 h intervals. In addition, some infected larvae were sacrificed and processed at 144 h and 336 h after inoculation.

For histopathological analysis, infected and uninfected (control group) larvae were fixed by injecting 100 µl of buffered formalin with an insulin syringe into the last left proleg. Larvae were kept in 40 ml formalin at room temperature. Subsequently, each larva was cut into two parts along the midline. Both parts were kept and processed for histology (Sakura Tissue-Tek VIP), embedded in paraffin wax (Thermo Scientific HistoStar embedding center), sectioned at 4 µm using a Leica RM2135 microtome, and then stained with haematoxylin and eosin (H&E), and Ziehl-Neelsen (ZN) stain, a specific stain for acid fast bacteria. The tissue slides were examined under a light microscope (Nikon Eclipse 80i) and photographed using a digital camera (Nikon DS-Ri1) attached to a computer equipped with NIS-Elements BR imaging software.

For TEM, at least 10 larvae per time-point (0, 24, 48, 72, 96, 144, 366 h) per group were used for haemolymph extraction. Haemolymph was collected from the haemocoel by puncturing the larvae in the last left pro-leg using a sterile needle. Haemolymph was carefully squeezed out of the larvae by applying gentle pressure, pooled into 50 ml of 1% formaldehyde and kept at room temperature for 2 h. Cells were then centrifuged and the cell pellet was re-suspended in 4% glutaraldehyde buffer and stored at 2–8C. An aliquot of the cells was then placed onto positively charged slides for ZN staining. The remaining cells were post-fixed with 1% osmium tetroxide (OsO4). After dehydration and embedding of the samples, ultra-thin (70–90 nm) sections were mounted on to grids and stained with uranyl acetate (0.5% w/v) and lead citrate (3% w/v) prior to examination using a Tecnai bioTWIN transmission electron microscope.

## Abbreviations

ADC: Albumin Dextrose Catalase
CFU: Colony Forming Units
*G. mellonella:*: *Galleria mellonella*
H&E: Haematoxylin and Eosin
LTBI: Latent tuberculosis infection
*M. bovis:*: *Mycobacterium bovis*
*M. bovis* BCG *lux:*: *Mycobacterium bovis* BCG *lux*
*M. tuberculosis:*: *Mycobacterium tuberculosis*
OADC: Oleic Acid, Albumin, Dextrose, Catalase
OsO_4_: Osmium tetroxide
PBS: Phosphate Buffered Saline
RLU: Relative Light Units
TAGs: Triacylglycerides
TEM: Transmission Electron Microscopy
ZN: Ziehl Neelsen
