# Supplementary material for: Galleria mellonella - a novel infection model for the Mycobacterium tuberculosis complex
Source: Virulence. 2018 Aug 1;9(1):1126–37. doi: 10.1080/21505594.2018.1491255 (PMC6086298; doi:10.1080/21505594.2018.1491255)
Supplement: Supplemental Material [file kvir-09-01-1491255-s001.zip › Li et al Supplementary Table 1.docx]

| **Group** |  | **Time (h)** | | | |
| --- | --- | --- | --- | --- | --- |
|  |  | **24** | **48** | **72** | **96** |
| **Dose (BCG)** | **Survival Run 1 (n)** | 14 | 8 | 0 | 0 |
| **2x10^7 CFU** | **Survival Run 2 (n)** | 19 | 9 | 3 | 1 |
|  | **Survival Run 3 (n)** | 14 | 8 | 5 | 0 |
|  | **Total survived (n)** | 47 | 25 | 8 | 1 |
|  | **Total number (n)** | 60 | 60 | 60 | 60 |
|  | **% survival** | 78 | 42 | 13 | 2 |
|  | **% dead** | 22 | 58 | 87 | 98 |
|  | **Median survival (n)** | 14 | 8 | 3 | 0 |
|  |  |  |  |  |  |
|  |  | **Time (h)** | | | |
|  |  | **24** | **48** | **72** | **96** |
| **Dose (BCG)** | **Survival Run 1 (n)** | 18 | 16 | 12 | 8 |
| **1x10^7 CFU** | **Survival Run 2 (n)** | 17 | 17 | 15 | 12 |
|  |  |  |  |  |  |
|  | **Total survived (n)** | 35 | 33 | 27 | 20 |
|  | **Total number (n)** | 40 | 40 | 40 | 40 |
|  | **% survival** | 88 | 83 | 68 | 50 |
|  | **% dead** | 13 | 18 | 33 | 50 |
|  | **Median survival (n)** | 17.5 | 16.5 | 13.5 | 10 |
|  |  |  |  |  |  |
|  |  | **Time (h)** | | | |
|  |  | **24** | **48** | **72** | **96** |
| **Dose (BCG)** | **Survival Run 1 (n)** | 20 | 20 | 20 | 20 |
| **1x10^6 CFU** | **Survival Run 2 (n)** | 20 | 20 | 20 | 20 |
|  | **Survival Run 3 (n)** | 20 | 20 | 20 | 20 |
|  | **Total survived (n)** | 60 | 60 | 60 | 60 |
|  | **Total number (n)** | 60 | 60 | 60 | 60 |
|  | **% survival** | 100 | 100 | 100 | 100 |
|  | **% dead** | 0 | 0 | 0 | 0 |
|  | **Median survival (n)** | 20 | 20 | 20 | 20 |
|  |  |  |  |  |  |
| **Group** |  | **Time (h)** | | | |
|  |  | **24** | **48** | **72** | **96** |
| **Dose (BCG)** | **Survival Run 1 (n)** | 10 | 10 | 10 | 10 |
| **1x10^5 CFU** | **Survival Run 2 (n)** | 10 | 10 | 10 | 10 |
|  | **Survival Run 3 (n)** | 10 | 10 | 10 | 10 |
|  | **Total survived (n)** | 30 | 30 | 30 | 30 |
|  | **Total number (n)** | 30 | 30 | 30 | 30 |
|  | **% survival** | 100 | 100 | 100 | 100 |
|  | **% dead** | 0 | 0 | 0 | 0 |
|  | **Median survival (n)** | 10 | 10 | 10 | 10 |
|  |  |  |  |  |  |
|  |  | **Time (h)** | | | |
|  |  | **24** | **48** | **72** | **96** |
| **PBS control** | **Survival Run 1 (n)** | 10 | 10 | 10 | 10 |
|  | **Survival Run 2 (n)** | 10 | 10 | 10 | 10 |
|  | **Survival Run 3 (n)** | 10 | 10 | 10 | 10 |
|  | **Total survived (n)** | 30 | 30 | 30 | 30 |
|  | **Total number (n)** | 30 | 30 | 30 | 30 |
|  | **% survival** | 100 | 100 | 100 | 100 |
|  | **% dead** | 0 | 0 | 0 | 0 |
|  | **Median survival (n)** | 10 | 10 | 10 | 10 |
|  |  |  |  |  |  |
|  |  | **Time (h)** | | | |
|  |  | **24** | **48** | **72** | **96** |
| **Pricked control** | **Survival Run 1 (n)** | 10 | 10 | 10 | 10 |
|  | **Survival Run 2 (n)** | 10 | 10 | 10 | 10 |
|  | **Survival Run 3 (n)** | 10 | 10 | 10 | 10 |
|  | **Total survived (n)** | 30 | 30 | 30 | 30 |
|  | **Total number (n)** | 30 | 30 | 30 | 30 |
|  | **% survival** | 100 | 100 | 100 | 100 |
|  | **% dead** | 0 | 0 | 0 | 0 |
|  | **Median survival (n)** | 10 | 10 | 10 | 10 |

**Supplementary Table 1. Effect of varying inoculum dose of *M. bovis* BCG *lux* on the survival of *G. mellonella* larvae following incubation at 37 ̊C for 96 h.**

Healthy larvae (n=≥ 10 per group) infected with differing inoccula of *M. bovis* BCG *lux* (up to 2 x 10 ^7^ CFU/larva) were examined for survival by response to touch at 24 h intervals. Control groups (n=10 larvae per group) included an uninfected group inoculated with 10 µl PBS-Tween 80, and a ‘pricked’ larval group (empty needle injection). Larvae were incubated at 37 ̊C in the dark and monitored on a daily basis. Larvae that did not show any movement in response to touch were considered dead. Numbers of larvae that were alive at each time-point for each experimental run are recorded. Data are pooled from at least two independent experiments.
