# Supplementary material for: Galleria mellonella - a novel infection model for the Mycobacterium tuberculosis complex
Source: Virulence. 2018 Aug 1;9(1):1126–37. doi: 10.1080/21505594.2018.1491255 (PMC6086298; doi:10.1080/21505594.2018.1491255)
Supplement: Supplemental Material [file kvir-09-01-1491255-s001.zip › Li et al. Supplementary Figure 1.pptx]

## Slide 1
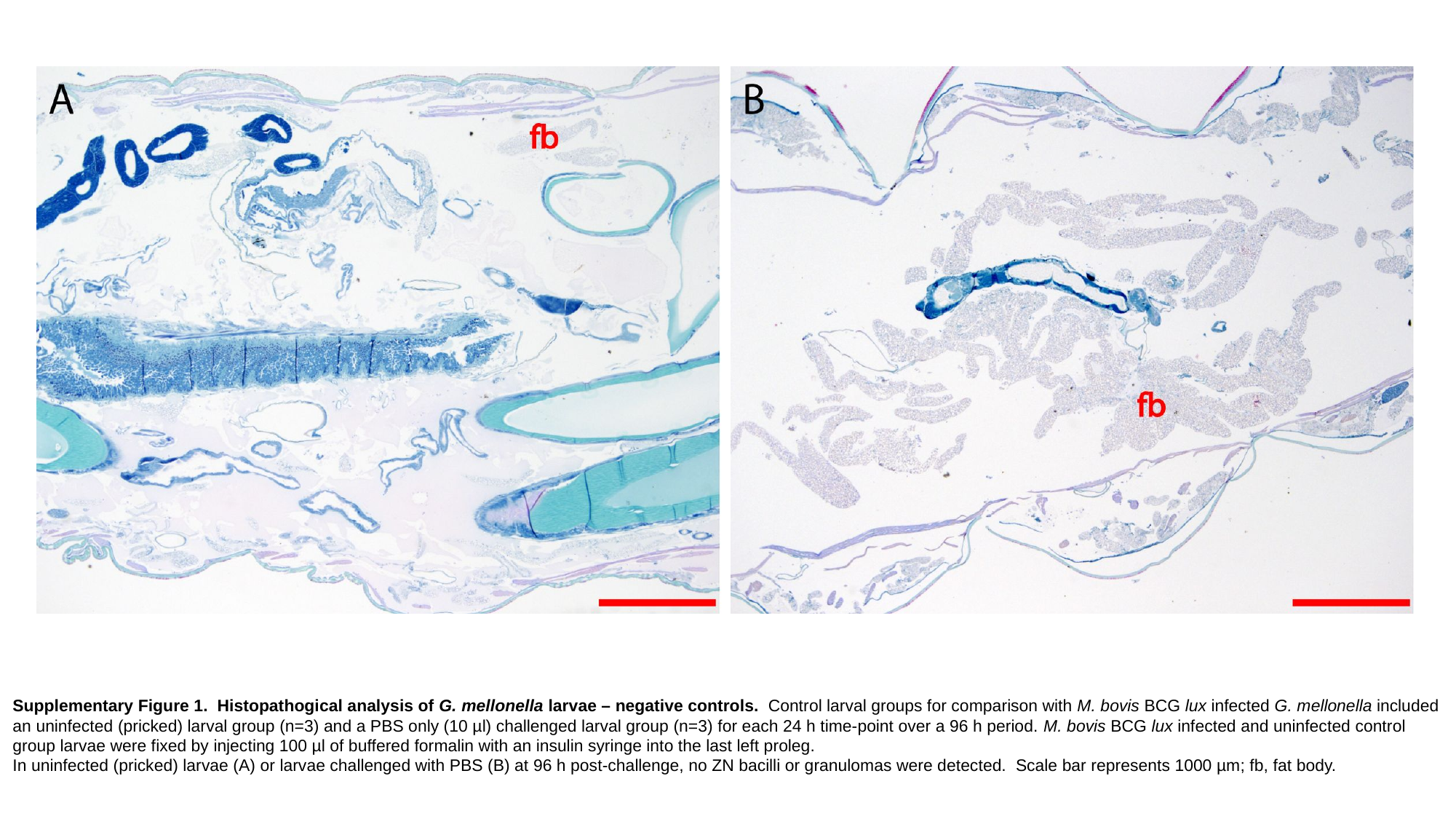

Supplementary Figure 1. Histopathogical analysis of G. mellonella larvae – negative controls. Control larval groups for comparison with M. bovis BCG lux infected G. mellonella included an uninfected (pricked) larval group (n=3) and a PBS only (10 µl) challenged larval group (n=3) for each 24 h time-point over a 96 h period. M. bovis BCG lux infected and uninfected control group larvae were fixed by injecting 100 µl of buffered formalin with an insulin syringe into the last left proleg.
In uninfected (pricked) larvae (A) or larvae challenged with PBS (B) at 96 h post-challenge, no ZN bacilli or granulomas were detected. Scale bar represents 1000 µm; fb, fat body.
