# Supplementary material for: Galleria mellonella - a novel infection model for the Mycobacterium tuberculosis complex
Source: Virulence. 2018 Aug 1;9(1):1126–37. doi: 10.1080/21505594.2018.1491255 (PMC6086298; doi:10.1080/21505594.2018.1491255)
Supplement: Supplemental Material [file kvir-09-01-1491255-s001.zip › Li et al. Supplementary Figure 2.pptx]

## Slide 1
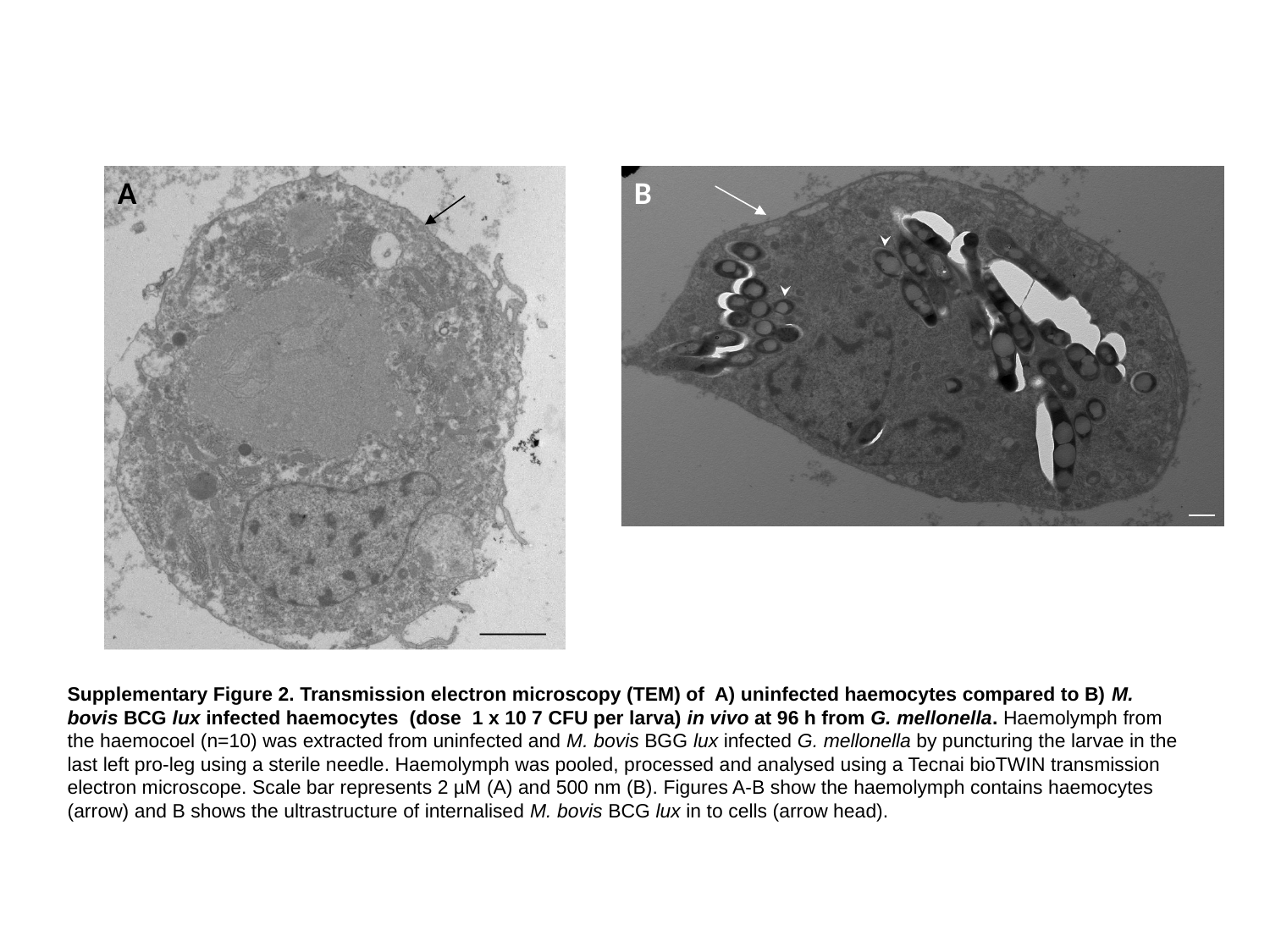

A
B
Supplementary Figure 2. Transmission electron microscopy (TEM) of A) uninfected haemocytes compared to B) M. bovis BCG lux infected haemocytes (dose 1 x 10 7 CFU per larva) in vivo at 96 h from G. mellonella. Haemolymph from the haemocoel (n=10) was extracted from uninfected and M. bovis BGG lux infected G. mellonella by puncturing the larvae in the last left pro-leg using a sterile needle. Haemolymph was pooled, processed and analysed using a Tecnai bioTWIN transmission electron microscope. Scale bar represents 2 µM (A) and 500 nm (B). Figures A-B show the haemolymph contains haemocytes (arrow) and B shows the ultrastructure of internalised M. bovis BCG lux in to cells (arrow head).
